# Single-Incision Laparoscopic Cholecystectomy: Initial Report From the Turks and Caicos Islands

**DOI:** 10.7759/cureus.14891

**Published:** 2021-05-07

**Authors:** Shamir O Cawich, Philip E Burgess, Deonne Ranglin-Robinson, Rufus Ewing

**Affiliations:** 1 Surgery, University of the West Indies, St. Augustine, TTO; 2 Surgery, Cheshire Hall Medical Center, Providenciales, TCA; 3 Anaesthesia, Cheshire Hall Medical Center, Providenciales, TCA

**Keywords:** cholecystectomy, caribbean, single incision, laparoscopic, gallbladder, sils

## Abstract

There has been no prior report of single-incision laparoscopic surgery (SILS) from the Caribbean island of Turks and Caicos. We report our initial experience with SILS cholecystectomy to show that SILS in this environment is feasible with minimal change to the existing hardware. It is a safe alternative to conventional multi-trocar laparoscopic cholecystectomy in this setting.

## Introduction

Caribbean surgeons accept conventional multiport laparoscopy (MPL) as the gold standard operation to treat benign gallbladder disease. The first single-incision laparoscopic surgery (SILS) cholecystectomy in the Caribbean was performed in 2009 by Cawich et al. [1] in an attempt to further reduce the invasiveness of the procedure. The SILS approach has gained popularity in the Caribbean, but there has been no prior report from the Turks and Caicos Islands (TCI). This report details our initial experience with SILS cholecystectomy in the TCI.

## Case presentation

A 41-year-old man presented with a confirmed diagnosis of acute cholecystitis and was managed conservatively. He was offered cholecystectomy through an SILS approach on an elective basis. Abdominal ultrasound confirmed cholelithiasis, and there was no clinical, sonographic, or biochemical evidence of choledocholithiasis.

**Figure 1:**
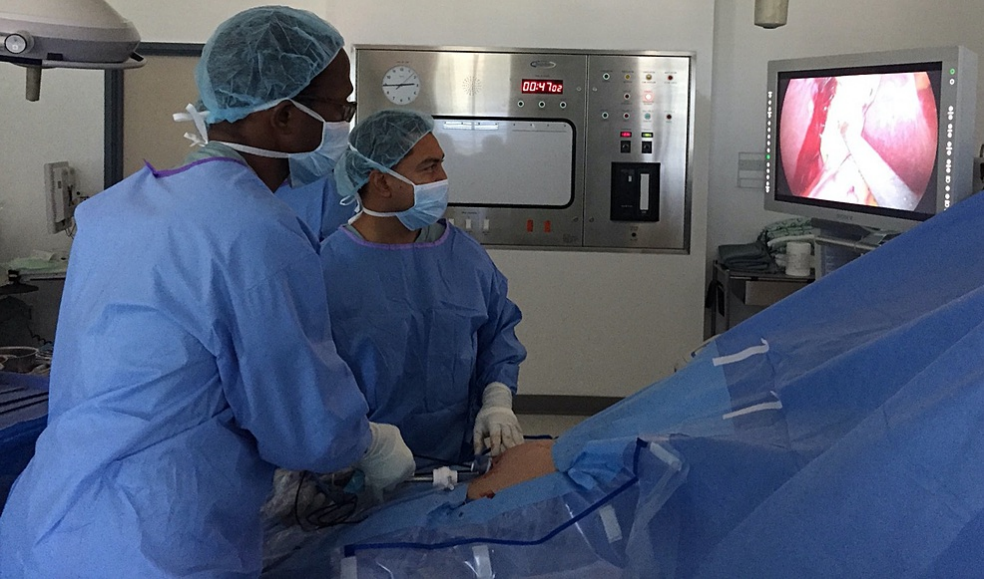
Positioning of surgical team during SILS. A view of the operating position with the surgeon between the legs using articulating instruments and a standard 30-degree laparoscope introduced in an SILS port. SILS, single-incision laparoscopic surgery

The patient was prepared for anesthesia and taken to the operating theater. No antibiotic prophylaxis was administered. Hasson’s technique was used to gain access to the peritoneal cavity through a 2.5-cm vertical umbilical incision. We used an SILS port® (Covidien Inc., Norwalk, CT, USA) inserted through the umbilical incision to create a 12-mmHg pneumoperitoneum. A standard 30-degree laparoscope was introduced to establish vision. The surgeon stood between the legs and introduced two articulating instruments into the 5-mm working ports to commence dissection (Figure 1).

**Figure 2:**
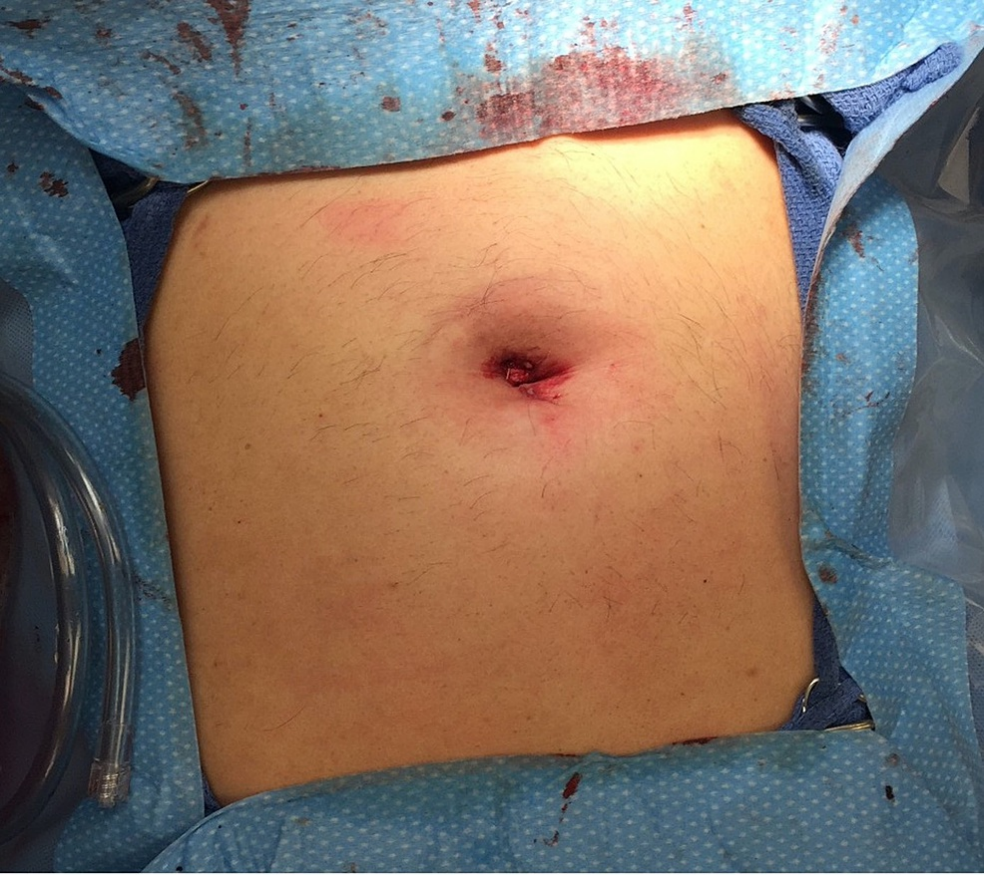
Umbilical incision used for SILS cholecystectomy. Post-operation, a single incision is seen at the umbilicus that will not be visible once the wound is fully healed. SILS, single-incision laparoscopic surgery

A 5-mm traumatic grasper was used to manipulate the gallbladder infundibulum, and Calot’s triangle was dissected using a combination of electrocautery and a Maryland dissector. Using cautery dissection in a retrograde fashion, biliary structures were identified and Strasberg’s critical view was achieved. We used 5-mm clips to ligate the cystic duct prior to transection. The gallbladder was dissected from the liver bed using electrocautery. Once detached, the gallbladder was extracted from the abdomen through the fascial incision used to introduce the SILS port. Fascial closure was achieved with 1/0 Prolene sutures (Ethicon Inc., West Somerville, NJ, USA) and skin was closed with 4/0 Monocryl sutures (Ethicon Inc.). The operation lasted 45 minutes, and no complications were recorded. He was discharged within 12 hours of the procedure with oral analgesia and had an uneventful recovery. The patient was seen in the surgical clinic six weeks post-operation and expressed satisfaction with the aesthetic outcome (Figure 2).

## Discussion

The TCI are a group of islands in the northern West Indies, approximately 650 miles south-east of Florida, with a population of 55,926 at the last census [2]. The Government of TCI provides subsidized healthcare to all legal residents through a network of public health clinics across the islands that are funded by a National Health Insurance Plan. Patients with gallbladder disease who require surgical care are referred to a single tertiary referral facility in the city of Providenciales. This facility is staffed by general surgeons with experience in conventional MPL surgery. The first laparoscopic operation in the TCI was a conventional MPL cholecystectomy performed by R. Ewing and colleagues at the Myrtle Rigby Complex in 2003. Since then, MPL has gained popularity on the TCI.

In an attempt to advance the repertoire of laparoscopic procedures offered in the TCI, the facility organized a laparoscopic workshop in the year 2018 funded by Medtronic Latin America, during which local surgeons were proctored in SILS. This report details the first SILS cholecystectomy to be performed on the TCI and shows that the operation is feasible and can be performed safely in this setting with appropriate proctorship.

This comes two decades after Navarra et al. [3] reported the first SILS cholecystectomy in 1997. Similar to Navarra et al.’s experience [3], the SILS technique was slow to gain momentum in the Caribbean after the first reported case in 2009 [1]. Our report now comes two decades after the original descriptions, but it is notable that SILS is only practiced in six Caribbean countries, inclusive of TCI [4] - a testament to the slow adoption of this technique by Caribbean surgeons. The technique is now gaining momentum largely due to the development of instruments specifically engineered for SILS, availability of training/proctorship, and support by industry.

Currently, SILS is accepted as an alternative to conventional MPL for cholecystectomy. To date, six randomized controlled trials [5-10] and one meta-analysis [11] comparing SILS and conventional MPL cholecystectomy have been published. In the published meta-analysis, Markar et al. [11] reported that both techniques had equivalent morbidity, post-operative pain scores, and duration of hospitalization. The individual trials reported that SILS brought greater patient satisfaction [5,10], improved quality of life [6], and better cosmesis [5-10]. The available data demonstrate that SILS is a feasible and safe alternative to MPL cholecystectomy.

One obstacle we anticipated was a long operating time that would lengthen waiting lists for surgery. There is no prior report from the TCI for comparison, but the operative time for our first SILS cholecystectomy was comparable to that for conventional MPL cholecystectomies in the Caribbean literature [12-16]. This should not be an obstacle to incorporating SILS into the local repertoire of operations.

Initially, local surgeons were apprehensive that the limited vision and anticipated instrument clashes would pose a barrier to this procedure. However, the articulating instruments have obviated this concern, allowing good ergonomics and reducing instrument clashes. These specialized instruments increase the cost associated with the procedure. One means for cost containment is to perform SILS procedures through the direct fascial puncture technique [17], but we acknowledge that this requires further training and experience. In this regard, we believe that the SILS port and articulating instruments provide good value for money as they can be rapidly assimilated by an accomplished general surgeon with laparoscopic experience.

This report adds to the existing data in support of SILS cholecystectomies in the Caribbean. This is important because conventional laparoscopic surgeons in the Caribbean continue to argue that the SILS approach is not well suited to the Caribbean healthcare environment [4,18,19], reminiscent of the resistance that open surgeons had for conventional MPL in the 1990s. This barrier will only be overcome when more data are available to prove that SILS is feasible and safe in the Caribbean setting. We acknowledge that SILS operations require advanced surgical skill sets to adapt to counterintuitive hand movements, instrument collision, and restricted movements of instruments through one entry point. In addition to proctorship, local surgeons may further hone these skills by extra-corporeal training with simulators [18].

## Conclusions

Incorporating SILS into the local surgical armamentarium is feasible with minimal change to the existing hardware. It is a safe alternative to conventional multi-trocar laparoscopic cholecystectomy in this setting.
